# The First Random Observational Survey of Barrier Gestures against COVID-19

**DOI:** 10.3390/ijerph18199972

**Published:** 2021-09-22

**Authors:** Véronique Renault, Marie-France Humblet, Gianni Parisi, Anne-Françoise Donneau, Fabrice Bureau, Laurent Gillet, Sébastien Fontaine, Claude Saegerman

**Affiliations:** 1 Fundamental and Applied Research for Animal Health (FARAH) Centre, Research Unit in Epidemiology and Risk Analysis Applied to Veterinary Sciences (UREAR-ULiege), Faculty of Veterinary Medicine, University of Liege, 4000 Liege, Belgium; vrenault@uliege.be (V.R.); gianni.parisi@uliege.be (G.P.); 2 Unit Biosafety, Department for Occupational Protection and Hygiene, Biosecurity and Environmental Licences, University of Liege, 4000 Liege, Belgium; mfhumblet@uliege.be; 3 Biostatistics Unit, Public Health Department, University of Liege, 4000 Liege, Belgium; afdonneau@uliege.be; 4 Laboratory of Cellular and Molecular Immunology, GIGA Institute, University of Liege, 4000 Liege, Belgium; fabrice.bureau@uliege.be; 5 Immunology-Vaccinology, Fundamental and Applied Research for Animal Health (FARAH) Centre, Faculty of Veterinary Medicine, University of Liege, 4000 Liege, Belgium; L.Gillet@uliege.be; 6 Department of Social Sciences, Faculty of Social Sciences, University of Liege, 4000 Liege, Belgium; Sebastien.Fontaine@uliege.be

**Keywords:** COVID-19, observation, compliance, barrier gestures, Belgium, student, prevention, barometer

## Abstract

In the context of COVID-19 in Belgium, face-to-face teaching activities were allowed in Belgian universities at the beginning of the 2020–2021 academic year. Nevertheless, several control measures were established to control COVID-19 transmission on the campuses. To ensure compliance with these measures, a random observational survey, based on five barrier gestures, was implemented at the University of Liege (greetings without contact, hand sanitisation, following a one-way traffic flow, wearing a mask and physical distancing). Each barrier gesture was weighted, based on experts’ elicitation, and a scoring system was developed. The results were presented as a diagram (to identify the margin of improvement for each barrier gesture) and a risk management barometer. In total, 526 h of observations were performed. The study revealed that some possible improvements could be made in the management of facilities, in terms of room allocation, the functionality of hydro-alcoholic gel dispensers, floor markings and one-way traffic flow. Compliance with the barrier gestures reached an overall weighted score of 68.2 (between 0 and 100). Three barrier gestures presented a lower implementation rate and should be addressed: the use of hydro-alcoholic gel (particularly when exiting buildings), compliance with the traffic flow and the maintenance of a 1.5 m physical distance outside of the auditoriums. The methodology and tool developed in the present study can easily be applied to other settings. They were proven to be useful in managing COVID-19, as the barometer that was developed and the outcomes of this survey enabled an improved risk assessment on campuses, and identified the critical points to be addressed in any further public health communication or education messages.

## 1. Introduction

COVID-19 represents the largest pandemic of the century, with more than 229 million confirmed cases worldwide at the end of June 2021 (https://covid19.who.int/ 21st July 2021). Belgium reported its first case on 4 February 2020 and the virus began to actively circulate in the country at the beginning of March 2020. The federal authorities implemented the first lockdown in mid-March in order to prevent the possible overwhelm of the healthcare system. By the end of April, the situation was stabilised and the lockdown was partially lifted. Nevertheless, the risk of a second wave in the autumn—due to people returning from summer holidays, the more favourable climatic conditions for COVID-19 and the return of students in schools and on campus—was perceived as high by the scientific community and the authorities. The reopening of the Universities in September 2020 represented a major risk for COVID-19 transmission, as face-to-face teaching activities require the gathering of a large number of persons in a closed environment [1,2]. In addition, as the majority of students in Belgium live in group housing and use public transport, they represent a population that is at higher risk of being in contact with infected persons and, thus, to contaminate others on site, as well as in their families when they return home on weekends. The Belgian education authorities defined a colour code corresponding to the risk level (i.e., green, yellow, orange and red). Each colour corresponds to the specific teaching conditions one must implement during the 2020–2021 academic year, as shown in Table A1. The 2020–2021 academic year began under the yellow code (i.e., low risk). Face-to-face teaching activities were allowed, with a reduced number of students and under the conditions issued by the Wallonia-Brussels Federation [3]. These conditions include: (i) the respect of a quarantine in cases of high risk contact or a positive test result; (ii) limitations to gathering (e.g., restricted social bubbles, forbidden indoor sports and competitions); (iii) wearing a mask indoors; (iv) maintaining a 1.5 m physical distance; (v) ensuring regular hand sanitisation; (v) following a one-way traffic flow. In addition to such measures, the University of Liege implemented a large-scale COVID-19 screening of university members (staff and students) using saliva samples and RT-qPCR testing.

The compliance of university staff and students with the measures was necessary for the control program to be effective, and the university authorities implemented an intensive communication campaign in that regard. Nevertheless, the compliance of the university members with these different measures was voluntary and difficult to control. Previous experiences of pandemics showed that there was a low level of compliance from the population in adhering to restrictive measures such as isolation [4], highlighting the need to use social and behavioural sciences and studies in order to better manage the risks and improve the compliance of the population with the needed measures [5]. From 28 September 2020, in addition to the compliance study, a longitudinal online survey assessed the reported implementation and acceptability of the barrier measures in order to adapt the communication to the university community. This acceptability survey was completed on a voluntary basis and mainly reached university members who participated in a salivary screening. The survey on acceptability showed that students’ perception of COVID-19 severity and the benefits of measures was lower (compared to the staff); furthermore, a lower sense of health responsibility was reported in non-medical faculties, compared to the faculties of medicine and veterinary medicine (unpublished data, Renault et al., 2021). Due to these beliefs, the students were less likely to adopt, and effectively implement, the different measures, as reported in another study stressing the lower compliance of young adults [6].

The present random observational survey aimed to assess the actual implementation of five barrier gestures during face-to-face teaching activities on a university campus, in order to monitor their level of implementation. These data fed an automated barometer used by the university authorities to trigger corrective actions addressing the weak points of the control program.

## 2. Materials and Methods

### 2.1. Survey Methodology

Five barrier gestures were observed in the auditoriums, the hallways and the restaurants of the four campuses of Liege University: (1) greetings without contact, (2) hand sanitisation (hand rubbing with hydro-alcoholic gel) when entering and exiting the different areas, (3) following a one-way traffic flow, (4) wearing a mask properly, (5) maintaining a 1.5 m physical distance between students in the auditoriums and outside.

A data collection tool was developed for the enumerators to record their data on paper. Afterwards, they uploaded the results of their observations in Epicollect5. This operation was performed while still on site in order to record the precise geographical coordinates of the observation’s location and an immediate overview of the situation. The data analysis could, therefore, be performed when needed, to adjust the control strategy based on the real-time monitoring of the field conditions [7].

Thirty-five students were recruited and trained as enumerators in order to properly classify the observed behaviours as ‘compliant’ or ‘non-compliant’ for each barrier gesture, and to properly use Epicollect5. Each enumerator was assigned three to four randomly selected observation sites per week. The observation periods were selected based on the occupational schedule of auditoriums.

Each enumerator received a document detailing the course of an observation session. Each session started with the identification of the most appropriate observation spot and a check for the presence of hydro-alcoholic gel and visible traffic flow directives (e.g., arrows on the ground or ‘no-entry’ signs on doors). If absent, their related barrier gesture (i.e., hand sanitisation at entry or exit and following a one-way traffic flow) was not observed. Each barrier gesture was observed over a 10 min-period in order to record individuals’ compliance with these gestures. Based on the density of persons to be observed, the enumerator either observed and recorded the compliance (N occurrences) for 5 min and the non-compliance for 5 min, or recorded both the compliant and non-compliant behaviours together for 10 min (when the number of persons was low). The observation process was slightly different in auditoriums for physical distancing and mask wearing. To assess physical distancing, the enumerators recorded the number of students that left a chair unoccupied on both sides (compliant behaviours), as well as the total number of students. Regarding masks, they counted the number of students wearing masks correctly in a given number of seats. In both cases, the number of non-compliant behaviours was then calculated by subtracting the number of compliant observations from the total number of students observed. At the end of the observation period, the enumerators filled in the Epicollect5 survey and registered, for each barrier gesture, the number of compliant behaviours observed, the number of non-compliant behaviours observed and the total number of observations performed. The paper forms were stored for future cross-validation and recordkeeping. For the different barrier gestures, the percentage of compliance was then calculated as follows:

(1) $$\mathrm{Percentage}\;\mathrm{of}\;\mathrm{compliance}=\frac{\mathrm{Number}\;\mathrm{of}\;\mathrm{compliant}\;\mathrm{behaviours}\;\mathrm{observed}}{\mathrm{Total}\;\mathrm{number}\;\mathrm{of}\;\mathrm{behaviours}\;\mathrm{observed}}\times 100$$

Data were collected from week 39 to week 43, in 2020. The first four weeks of observations (weeks 39 to 42) were implemented in the yellow code context, while the code changed to orange by week 43 (reduced face-to-face activities and number of students per training session). Observations were suspended on October 26th (week 44) due to the suspension of all face-to-face activities on site (red code) ordered by the Government (Table A1). The proportion of students who tested positive for COVID-19 (as part of the salivary testing program) during this same period reached 2%, 4%, 5%, 9% and 10% during week 40, 41, 42, 43 and 44, respectively. On week 39, 0% of students tested positive, but only two students were tested (unpublished data from the University of Liege).

### 2.2. Data Analysis

Data were extracted from Epicollect5 and cleaned. Five percent of the observation forms extracted from Epicollect5 were randomly sampled and checked for conformity with the paper forms. The archived paper forms were also used to complete eventual missing or erroneous data in the Epicollect5 forms.

Statistical analyses were performed using Stata SE 14.2 (StataCorp, College Station, Texas, USA). A quantile regression allowed for the comparison of compliance rates between campuses and observation sites (auditoriums, restaurant/cafeteria, hallways and corridors), the weeks of observation (week 39 to 43) and the colour code periods (yellow or orange), as well as per building and auditorium capacity.

### 2.3. Expert Elicitation of Knowledge and Weighting System

In order to develop a scoring system taking into account the importance of the different barrier gestures in the control of COVID-19, an expert elicitation of knowledge was conducted to gather the opinions of 41 professionals with recognised scientific expertise and/or experience in the field of COVID-19. For guidance purposes, a letter explaining the purpose of the study accompanied the questionnaire that each expert had to fill out. The questionnaire was prepared as an Excel file. Each expert was contacted personally and responded individually to the questionnaire. Data generated by the elicitation were based on the individual values provided by experts in order to capture the degree of variability of expert knowledge. The elicitation was performed in week 6, year 2021. The elicited experts were asked to provide three types of information. First, they were asked to specify their main domain(s) of expertise and their number of year of activities. Secondly, they were requested to weight each barrier gesture in terms of its efficacy against the transmission of SARS-CoV-2. This relative weight was determined using the Las Vegas technique [8]. Briefly, experts had to distribute 100 points between the barrier gestures, according to their relative importance in the prevention of SARS-CoV-2 transmission. If all barrier gestures were considered to be of equivalent importance by experts, the same number of points was allocated to each one. Lastly, for each weight attributed to a barrier gesture, the expert provided a note of uncertainty from 0 (maximal uncertainty) to 5 (total certainty).

### 2.4. Calculation of an Overall Weighted Score and Visualisation as a Barometer

The overall score of compliance was calculated using an aggregation method that combined the level of compliance of all barrier gestures and their relative weights. These results provided an overall weighted score (OWS) of compliance for each barrier gesture and per expert:

OWS = ∑ OLi × Wi (2)

In this formula, OWS = overall weighted score for the compliance with all observed barrier gestures; OLi = compliance level for a specific barrier gesture (value between 0 and 1 of the beta distribution); Wi = weight of a specific barrier gesture (100 points distributed between all barrier gestures depending on their relative importance in the control of SARS-CoV-2 transmission). The OWS ranged from 0 to 100. Finally, the compliance with each barrier gesture was presented as a spider web and the OWS was integrated to a barometer for better visualization.

In addition, a stochastic model was developed to establish the compliance level with the recommended barrier gestures (score from 0 to 100, equivalent to the median percentage of ‘compliant’ observations). Their confidence interval was assessed with a beta distribution, Alpha 1 being equivalent to ‘the compliant observations minus 1’, and Alpha 2 being equivalent to ‘the non-compliant observations minus 1’. The spreadsheet model was designed in Microsoft Excel (Microsoft^®^ Office 2016, Redmond, WA, USA). The model was run for 1,000 iterations (Monte Carlo sampling) in @Risk version 7.6 (© Palisade Corporation, Ithaca, NY, USA).

### 2.5. Sensitivity Analysis

A sensitivity analysis was performed according to the same methodology developed by [9] to analyse the robustness of the expert elicitation and the putative influence of the composition of the expert panel in the weight of each gesture barrier. Briefly, the robustness of expert elicitation was tested using a selection of 10 different panels of 25 experts, randomly selected among the 38 available experts. The distribution of weights from each panel and for each gesture barrier were compared in order to verify if discrepancies occur or not between the 10 different panels, using a non-parametric Kruskal–Wallis test [10]. The null hypothesis was that the 10 panels are selected from the same population. If this hypothesis is verified, the weight for each gesture barrier will not be dependent on the composition of the expert panel.

## 3. Results

A total of 526 observation sessions were implemented over the research period (339 performed in auditoriums, 57 in restaurants or cafeterias, and 106 in hallways or corridors (Table 1)). In 24 cases, the selected auditorium was found to be empty, due to a change of schedule or a shift to distance teaching, with no alternative observation rooms nearby; thus, no observations were made. The capacity of the selected auditoriums ranged from 10 to 513 persons.

### 3.1. Compliance with Barrier Gestures

Specific conditions were required for students to better comply with barrier gestures. The enumerators had to record whether hydro-alcoholic gel dispensers were present and operational, if a traffic flow was clearly defined and, in restaurants and cafeterias, if floor markings were visible (Table 2). More than 75% of the places observed had an operational hydro-alcoholic gel dispenser. Nevertheless, traffic flow was not clearly specified in 46% of auditoriums, 37% of hallways and 23% of restaurants. In addition, no floor markings were visible in four restaurants/cafeterias out of the 18 observed (one in Arlon, one in the centre of Liege and two on the Sart-Tilman campus).

The compliance rates for the different barrier gestures reached 83% for ‘Greetings’, 44% for ‘Hand sanitisation’, 65% for ‘Traffic flow’, 79% for ‘Wearing a mask correctly’, 89% for ‘Physical distancing in auditoriums’ and 44% for ‘Physical distancing outside the auditoriums’ (Table 3). Wearing a mask that was not covering the nose (72%) and the lack of a mask (13%) were the most frequent reasons for incorrect mask wearing, as mentioned by the enumerators. For 24% of the observation sessions, 100% of the people observed were properly wearing a mask.

Based on the univariate quantile regression model, using the Sart-Tilman campus as the reference, the compliance with ‘Hand sanitisation’ and ‘One-way traffic flow’ was significantly lower in Gembloux and Arlon, and significantly higher in the Liege centre (Table 4a). Compliance with ‘greetings without contact’ was significantly lower, while compliance with ‘physical distance’ was significantly higher at the entrance to restaurants/cafeterias, compared to auditoriums (Table 4a). The frequency of correct mask wearing was significantly higher in auditoriums, compared to hallways, corridors and restaurants (Table 4b). Hand sanitisation was significantly lower for people exiting the places, compared to entering persons (Table 4c), with a median of 15.4% and 50%, respectively. The compliance rate was also influenced by the week of observation (Table 4d) and the security colour code (Table 4e). Indeed, the number of students wearing a mask correctly was significantly higher during weeks 42 and 43 (vs. week 39), while the compliance with hand sanitisation was significantly higher during the orange code period (week 43), in comparison to the yellow code period (week 39–42).

**Figure 1:**
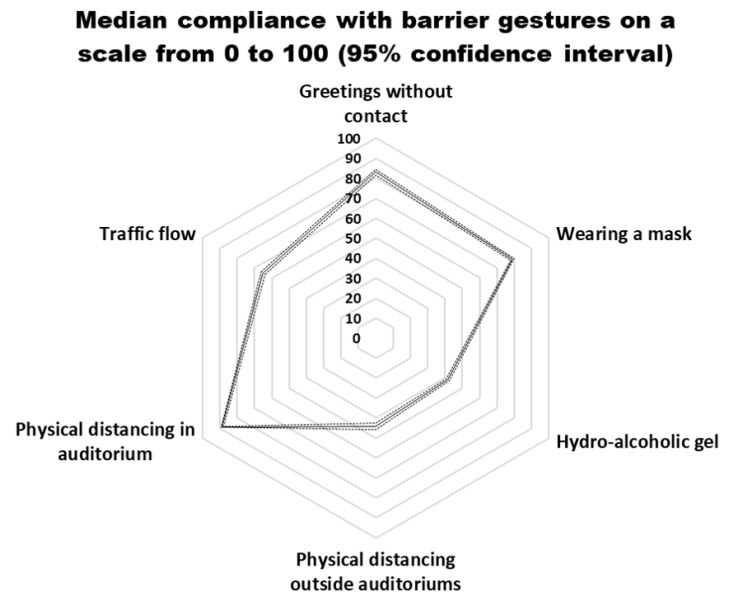
Median compliance with barrier gestures, scale from 0 to 100 (95% confidence interval).

The stochastic model provided compliance rates of 83% for ‘greetings without contact’, 42% for ‘Hand sanitisation’, 65% for ‘One-way traffic flow’, 79% for ‘Wearing mask’, 89% for ‘Leaving an empty chair on both sides in the auditoriums’ and 44% for ‘Maintaining a 1.5 m physical distance’ (Figure 1).

### 3.2. Calculation of an Overall Weighted Score and Translation as a Barometer

Thirty-seven experts participated in the elicitation (19 women and 18 men), corresponding to a 90% response rate (Table A2). Their area(s) of expertise were mainly public health, medicine, virology, infectious diseases (human and animal diseases, as well as zoonosis), epidemiology, biosecurity, vector-borne diseases, risk analysis, immunology and vaccinology, cellular and/or molecular biology and One Health.

**Figure 2:**
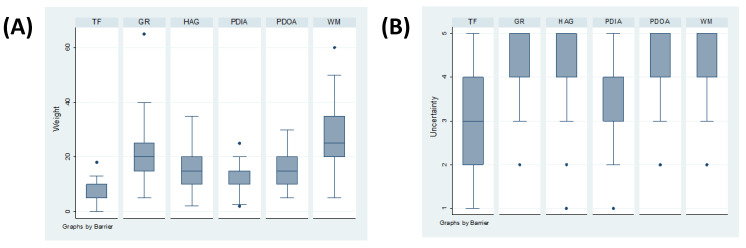
Expert opinion on the weight of the different barrier gestures (**A**) and their degree of certainty (**B**). TF: traffic flow, GR: greetings without contact, HAG: usage of hydro-alcoholic gel (hand sanitisation), PDIA: physical distancing inside the auditoriums, PDOA: physical distancing outside the auditoriums, WM: wearing a mask.

The importance of barrier gestures ranged from 0 (for compliance with traffic flow) to 65 (for compliance with greeting without contact). The highest weights were obtained for ‘Wearing masks’ and ‘Greetings without contact’ (Figure 2A). The median level of certainty mentioned by the experts was, overall, equal or above three (scale from 0 to 5) for all barrier gestures (Figure 2B).

**Figure 3:**
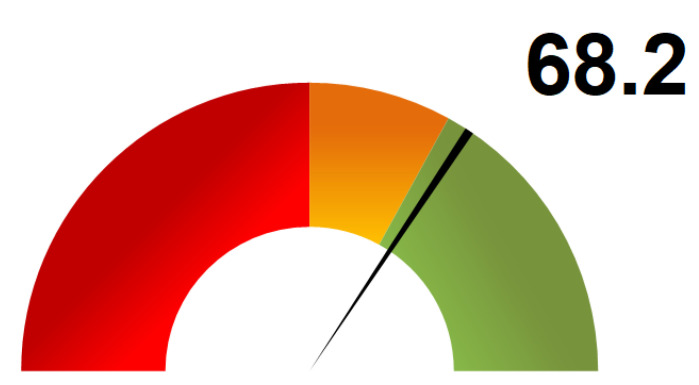
Overall weighted score (range: 0 to 100). The scale was arbitrarily divided into three parts: red—overall weighted score less than 50; yellow—overall weighted score between 50 and 66; green—overall weighted score above 66.

**Figure A1:**
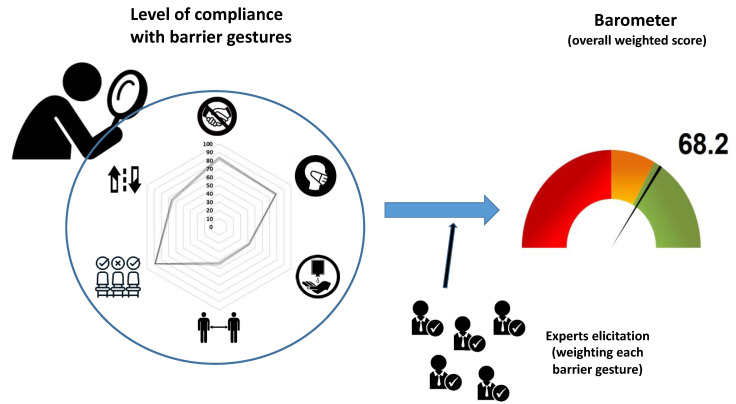
Graphical abstract presenting the feeding of the risk management barometer.

The overall weighted score (OWS) obtained is 68.2, on a range from 0 to 100 (Figure 3 and Figure A1).

### 3.3. Sensitivity Analysis

According to the sensitivity analysis based on the Kruskal–Wallis test, we confirmed the null hypothesis that all 10 panels of experts were selected from the identical population with the same median (Table 5). Indeed, the weight was not dependent on the composition of the panel of experts.

## 4. Discussion

To the best of our knowledge, this is the first random observational study of barrier gestures in relation to the COVID-19 pandemic. It was implemented over the four campuses of the University of Liege during a five-week period, from the start of the academic year to the suspension of face-to face teaching activities. The results are of key importance to assess the level of compliance lof staff and students with barrier gestures, and to adapt the communication plan and control measures of University of Liege, based on the observations and the main weaknesses of the control program.

The robustness of the expert elicitation (i.e., absence of influence of the composition of the panel of experts) regarding the weight of each gesture barrier was tested and confirmed using a sensitivity analysis.

The study revealed that there were some margins for improvement in the management of facilities in order to promote an enabling environment. Indeed, teaching facilities were not always adapted, either to the number of students or to the establishment of a traffic flow (e.g., classrooms with only one door/entrance). Floor markings were not visible in all restaurants/cafeterias to facilitate the maintenance of physical distances and establish a clear traffic flow. In addition, the study revealed that hydro-alcoholic gel dispensers were not available or operational in all buildings.

Three measures were complied with in more than 75% of observations. There is important room for improvements regarding the frequency of hand sanitisation (use of hydro-alcoholic gel), particularly when exiting buildings, the compliance with traffic flow, which, even when clearly visible, is not properly complied with, and compliance with physical distancing outside of the auditoriums. In addition, an online survey implemented over the same period at the University of Liege collected the reported compliance rate of these measures by the students (unpublished data, Renault et al., 2021). The reported rates showed some similar trends, with a high compliance for ‘Wearing masks’ (median frequency of 90%) and a lower compliance rate for ‘1.5 m-physical distance’ (median frequency of 60%). The reported frequency of ‘Hand sanitisation’ was higher (80%) than the observed rate. However, it was comparable to the observed rate at the entrance of the buildings. This might be linked to the fact that most people are less sensitised to the need for hand disinfection when exiting the premises, as the communication focused mainly on hand disinfection upon entering buildings. The absence of ‘direct physical contact’ was reported as compliant in 60% of cases, while the observations highlighted 83% of greetings without any contact. Such a difference can be explained by the fact that direct contact is unavoidable in contexts other than greetings, such as on public transportation and participation in practical activities, in which case, the compliance could have been reported as negative by the respondents, while the observation of greetings on site were mostly compliant.

The increased rate of correct mask wearing during week 42 and 43, and the increased compliance with hand sanitisation during the orange code, might be related to the increased risk perception during these periods. Indeed, the media relayed alerts regarding the increase in COVID-19 incidences and the start of a second wave. This hypothesis should be confirmed by further studies investigating the evolution of the risk perceptions over time. Focus group discussions have been planned in the different faculties in order to investigate this hypothesis and raise students’ awareness of the evolution of COVID-19 in the Institution. These focus group discussion will help to assess the influence of media headlines on behavior, and to discuss the level of student involvement at different times of the pandemic.

Most experts (*N* = 27) identified ‘Mask wearing’ as being the most important barrier gesture, with a high degree of certainty (median of five), which is in line with the findings of a previous study [1]. Compliance with a one-way traffic flow was ranked as the least important barrier gesture by 28 experts, although the degree of certainty was lower. It is encouraging to observe that the measures that were ranked as the most important by the experts (i.e., correct mask wearing and greetings without contact) were complied with the most by the students. Nevertheless, the 68.2% OWS shows that there is still a margin for improvement and the compliance with all barrier gestures is key to reducing disease transmission. It is, therefore, important to adjust the communication plan towards the students to increase their compliance with other measures.

An Excel tool was developed in order to automatically visualise the main results as a spider web and barometer by providing the number of compliant and non-compliant observations over time. As long as the observational study is implemented, such a tool can be used on a regular basis (e.g., each week) to monitor the situation and implement corrective measures if needed. It can also be effective for interventional studies in order to evaluate the impact of some specific communications on the different barrier gestures. As an example, the lack of functionality and/or the absence of hydro-alcoholic gel in several buildings that was observed at the beginning of the observational study was reported to the risk management group of the University of Liege and led to corrective measures. Nevertheless, in terms of management, improvements are still possible in terms of traffic flow and room allocation based on the number of students.

An integrated management system was adopted by the Institution for the next academic year, taking the form of a COVID-19 management cockpit; the barometer is one of 12 key indicators used to monitor COVID-19 evolution and take corrective actions when needed. In order to increase students’ compliance with barrier gestures and the institutional control program, a health communication plan was established, which includes regular mailings and visual posters reminding them of the barrier gestures. Communication will focus on the collective health responsibility, risk susceptibility and possible impact of COVID-19 at the population level, as these seem to be the determining factors related to COVID-19 specificities [11] (unpublished data, Renault V. et al. 2021).

## 5. Conclusions

The results of this survey are useful to properly manage the pandemic by identifying the existing weaknesses and reporting them to the managers for corrective measures to be put in place (e.g., the functionality of hydro-alcoholic gel dispensers was corrected based on our first observations). The barometer and spider web are two important monitoring tools that can be used in different settings for the continuous monitoring and better management of COVID-19, by identifying the public health risks and the topics that should be targeted in future health communications and prevention messages and tools. The observational survey should be pursued over the academic year in order to feed the tool, as well as to evaluate the eventual impact of some interventions (e.g., specific communication campaigns).

## Figures and Tables

**Table 1:** Distribution of observation sessions per campus and locations of observations.

Campus location	Hallways or Corridors	Auditoriums	Restaurants or Cafeterias	Total
Arlon	4	30	5	39
Gembloux	8	23	4	35
Liege Centre	30	139	15	184
Liege Sart-Tilman	64	171	33	268
Total	106	363	57	526

**Table 2:** Percentage of places without: (a) presence of hydro-alcoholic gel and (b) defined one-way traffic flow.

**(a) Presence of Hydro-Alcoholic Gel**						
		**Liege** **Sart-Tilman**	**Liege Centre**	**Gembloux**	**Arlon**	**Total**
**Place**	*N*	268	184	35	39	526
Auditoriums	363	16	23	4	20	18
Hallways or corridor	106	14	7	75	50	18
Restaurants or cafeterias	57	6	27	0	0	11
Total	526	15	21	20	21	17
**(b) Defined One-Way Traffic Flow**						
		**Liege Sart-Tilman**	**Liege Centre**	**Gembloux**	**Arlon**	**Total**
**Place**	*N*	268	184	35	39	526
Auditoriums	363	40	50	39	70	46
Hallways or corridors	106	38	23	50	100	37
Restaurants or cafeterias	57	18	13	0	100	23
Total	526	37	43	37	77	42

**Table 3:** Observed percentage of barrier gesture implementation and confidence interval.

Gesture	*N*	Observation with Respect	Percentage of Respect	Standard Error	Binomial Exact (95% Confidence Interval)	
Greetings	2768	2300	83	0.007	82%	84%
Hydro-alcoholic gel	8822	3868	44	0.005	43%	45%
Circulation flow	7335	4773	65	0.006	64%	66%
Wearing mask	10,856	8567	79	0.004	78%	80%
Physical distancing in auditoriums	7266	6452	89	0.004	88%	90%
Physical distancing out of auditorium	3587	1585	44	0.008	43%	46%

**Table 4:** Results of the univariate quantile regression models with significant differences based on: (a) sites, (b) place of observation, (c) observation of the entries or exits, (d) week, (e) code.

**(a) Site**						
	**Use of Hydro-Alcoholic Gel**		**Circulation Flow**		**Wearing Mask**	
Ref = Liege Sart-Tilman	Coeff	*p*-value	Coeff	*p*-value	Coeff	*p*-value
Liege Centre					5.6	0.014
Gembloux	−14.9	<0.001				
Arlon			−22.72727	0.009		
**(b) Place of observation**						
	**Greetings**		**Wearing mask**		**Physical distance**	
Ref = Auditorium	Coeff	*p*-value	Coeff	*p*-value	Coeff	*p*-value
Hallway/Corridor			−8.8	<0.001		
Restaurant or cafeteria	−11.11	0.005	−16	<0.001	52.9	<0.001
**(c) Observation of the entries or exits**						
	**Use of Hydro-Alcoholic Gel**					
	Coeff	*p*-value				
Exits	−34.6	<0.001				
						
**(d) Week**						
	**Wearing Mask**					
Ref = Week 39	Coeff	*p*-value				
Week 42	10.0	0.003				
Week 43	10	0.005				
						
**(e) Code**						
	**Use of Hydro-Alcoholic Gel**					
Ref = Yellow	Coeff	*p*-value				
Orange	12.381	0.017				

**Table 5:** Effect of the composition of the panel of experts on the weight of each gesture barrier contributing to SARS-CoV-2 transmission.

Barrier Gesture	Kruskal–Wallis Test	
	Chi-squared with 9 degrees of freedom	Probability
Greeting	2.316	0.985
Use of hydro-alcoholic gel	2.752	0.973
Traffic flow	2.362	0.984
Mask wearing	1.573	0.997
Physical distancing in auditoriums	4.043	0.909
Physical distancing outside auditoriums	2.805	0.972

## Data Availability

The data that support the findings of this study are available from the corresponding author upon reasonable request.

## Appendix A

**Table A1:** Colour codes in universities depending on the risk level regarding SARS-CoV2.

	Green	Yellow	Orange	Red
**Risk level**	No risk	Low risk	Moderate risk	High risk
**Interpretation of risk level**	Vaccine available and/or herd immunity. Contact may occur. Hand hygiene is still necessary	Limited spread of the virus. Contact is limited, but may occur depending on security conditions	Systematic transmission of the virus. Contacts are limited to the essentials and take place when risk factors are under control	Systematic transmission of the virus, contact is to be avoided as much as possible
**Occupancy of the premises**	Premises open and all services operational	Premises open Limitation to 75% of the maximum number of students possible Services ensured by respecting all hygienic measures	Premises open Limitation to 20% of the maximum number of students possible Services ensured by respecting all hygenic measures	Premises open with minimal services provided
**Teaching and evaluation activites**	Face-to-face activities possible	Face-to-face and distance-learning	Distance learning to be organised whenever possible	Distance learning only
**Group size ≤ 50**	No restriction	Physical distancing of 1 m. Mandatory mask wearing		Forbidden
**Groups of 521–200**	No restriction	Face covering and physical distancing of 1 m or occupation of 1 every 2 seats Professor without mask if a physical distance of 3 m is maintained	Face covering Occupation of 1 every 5 seats Professor without mask if a physical distance of 3 m is maintained	Forbidden
**Groups > 201**	No restriction	Face covering and physical distancing of 1 m or occupation of 1 every 2 seats Professor without mask if a physical distance of 3 m is maintained	Forbidden	Forbidden
**Movements**	Free	Unique traffic flow designated with arrows Mandatory mask wearing		
**Restaurants**	Free	Opened with physical distancing of 1.50 m. and outside settings to be prioritised Mandatory mask before and after eating		Not accessible

**Table A2:** Profiles of elicited experts.

Nr	Gender	Country	Year of Activity	Main Expertise
1	Female	Belgium	16	Biosecurity
2	Male	France	25	Virology
3	Male	Burkina Faso	5	Health ecology
4	Male	Belgium	22	Public health
5	Male	Spain	40	Epidemiology
6	Female	Ecuador	20	Prevention and control of diseases
7	Female	Belgium	13	Infectious and zoonotic diseases
8	Female	France	15	Virology
9	Female	Greece	3	Epidemiology
10	Male	Belgium	29	Emergency medicine
11	Female	Belgium	6	Medical sciences
12	Male	Belgium	20	Virology
13	Female	Belgium	16	Biosecurity
14	Female	Belgium	34	Prevention and health promotion
15	Female	France	37	Zoonoses
16	Female	Belgium	19	Biosecurity
17	Female	France	16	Epidemiology
18	Female	Belgium	12	Biosecurity
19	Male	Belgium	25	Infectious diseases
20	Female	France	15	Virology
21	Female	Belgium	29	Biosafety
22	Female	Ecuador	12	Ecology
23	Male	Belgium	19	Virology
24	Male	France	20	Virology
25	Male	France	25	Virology
26	Female	France	22	Wildlife/human/domestic animal interface
27	Male	Belgium	35	Immunology
28	Male	Cameroun	13	Control of diseases
29	Male	Belgium	19	Virology
30	Male	France	42	Immunology
31	Female	France	15	Health regulation
32	Male	Ecuador	20	Zoonoses
33	Female	Belgium	29	Control of infectious diseases
34	Female	France	30	Virology
35	Female	Luxemburg	13	Molecular epidemiology
36	Male	Belgium	40	Virology
37	Male	Canada	37	Biosecurity
38	Male	Belgium	32	Virology
